# Biomechanical Analysis of a Newly Developed Shape Memory Alloy Hook in a Transforaminal Lumbar Interbody Fusion (TLIF) *In Vitro* Model

**DOI:** 10.1371/journal.pone.0114326

**Published:** 2014-12-04

**Authors:** Xi Wang, Jing Xu, Yuexing Zhu, Jiukun Li, Si Zhou, Shunliang Tian, Yucheng Xiang, Xingmo Liu, Ying Zheng, Tao Pan

**Affiliations:** 1 Department of Orthopaedic Surgery, The Sixth Affiliated Hospital of Sun Yat-sen University, Guangzhou, PR China; 2 Department of Anatomy, Guilin Medical College, Guilin, PR China; 3 Department of Nutrition, Guangdong General Hospital, Guangzhou, PR China; University of Michigan, United States of America

## Abstract

**Objective:**

The objective of this biomechanical study was to evaluate the stability provided by a newly developed shape memory alloy hook (SMAH) in a cadaveric transforaminal lumbar interbody fusion (TLIF) model.

**Methods:**

Six human cadaveric spines (L1-S2) were tested in an *in vitro* flexibility experiment by applying pure moments of ±8 Nm in flexion/extension, left/right lateral bending, and left/right axial rotation. After intact testing, a TLIF was performed at L4-5. Each specimen was tested for the following constructs: unilateral SMAH (USMAH); bilateral SMAH (BSMAH); unilateral pedicle screws and rods (UPS); and bilateral pedicle screws and rods (BPS). The L3–L4, L4–L5, and L5-S1 range of motion (ROM) were recorded by a Motion Analysis System.

**Results:**

Compared to the other constructs, the BPS provided the most stability. The UPS significantly reduced the ROM in extension/flexion and lateral bending; the BSMAH significantly reduced the ROM in extension/flexion, lateral bending, and axial rotation; and the USMAH significantly reduced the ROM in flexion and left lateral bending compared with the intact spine (p<0.05). The USMAH slightly reduced the ROM in extension, right lateral bending and axial rotation (p>0.05). Stability provided by the USMAH compared with the UPS was not significantly different. ROMs of adjacent segments increased in all fixed constructs (p>0.05).

**Conclusions:**

Bilateral SMAH fixation can achieve immediate stability after L4–5 TLIF *in vitro*. Further studies are required to determine whether the SMAH can achieve fusion *in vivo* and alleviate adjacent segment degeneration.

## Introduction

Over the last 100 years, lumbar spinal fusion has evolved as the conventional treatment for infection, tuberculosis, fracture, malformation, arthritis, and degenerative disorders. Traditional posterior lateral fusion has been replaced by interbody fusion gradually, which provides solid fixation of spinal segments while maintaining a physical load-bearing capacity and proper disc height [1]. Interbody fusion involves reconstruction of the anterior column after disc removal, which is essential as 80% of compressive, torsion, and shear forces are transmitted through the anterior column [2].

Transforaminal interbody fusion (TLIF) was developed by Harms [3] as an alternative method for overcoming the risks and limitations associated with posterior lumbar interbody fusion (PLIF). TLIF using a unilateral approach allows preservation of the spinous processes and facets, and laminae on the contralateral side. This minimizes retraction on the thecal sac and neural elements, decreases the risk for a durotomy, limits possible neurological injury, and reduces intra-operative bleeding [4], [5]. The open TLIF procedure involves the stripping of the paravertebral muscles, which may negatively affect postoperative outcome. TLIF can also be operated using a minimally invasive technique [6], [7], which is thought to be favorable as it achieves similar surgical efficacy but is associated with reductions in operation time, blood loss, and hospital length of stay compared with open surgery.

TLIF involves fixation of adjacent vertebral bodies with implants to achieve fusion. Pedicle screw and rod fixation was popular, but widespread application is limited by postoperative complications and high costs [7], [8]. Both unilateral and bilateral pedicle screw-rod fixation in one- or two-segment lumbar spinal fusion have comparable complication rates [9]–[11]. Furthermore, if the primary operation with pedicle screw and rod fixation fails, the damaged posterior bony structure makes further rebuilding and stabilization of the spine more challenging [9], [10].

Since Buehler found nickel-titanium alloy had a shape memory effect in 1963 [11], nickel-titanium shape memory alloy (Ni-Ti SMA) implants have been successfully instrumented in orthopedics. Indeed, Ni-Ti SMA is a quick and easy implant that is used for surgical correction of scoliosis [12]. To date, and despite the unique mechanical properties and biocompatibility of SMA devices [13], [14], no reports document their use for lumbar fusion. Therefore, we evaluated the biomechanical stability offered by a newly developed SMA hook (SMAH) in a cadaveric TLIF model. We compared intact spines, a unilateral SMAH (USMAH), a bilateral SMAH (BSMAH), unilateral pedicle screws and rods (UPS), and bilateral pedicle screws and rods (BPS) in an *in vitro* flexibility experiment. We hypothesized that the semi-rigid fixation effect of the SMA construct would decrease the range of motion (ROM) of fixed segments compared to intact spines, and the ROM of adjacent segments, compared to fixation with pedicle screws and rods.

## Materials and Methods

### Ethics Statement

The cadavers were donated to the Department of Anatomy of Guilin Medical College for the purpose of teaching and preclinical research. Written informed consents from the donors or the next of kin were obtained before donation. Permission from the ethics committee of Guilin Medical College was obtained before the study. And the study was conducted according to the principles outlined in the Declaration of Helsinki.

### Specimen Preparation

Six male fresh-frozen human cadaver lumbosacral spines were used in this study (mean death age, 58.8 years; range, 45–67 years). Anterior-posterior and lateral radiographs of the spines confirmed the absence of any neoplastic disease, significant degeneration, fractures, or deformities [15], [16]. The bone mineral density (BMD) of each specimen was evaluated at L2–L5 by a computed tomography (CT) scan with a slice thickness of 2 mm. The mean BMD value of the 6 specimens was 1.08 g/cm^2^ ranging from 0.91 to 1.38 g/cm^2^. All specimens had normal bones without osteoporosis. Specimens were thawed at 4°C overnight. Subsequently, the paraspinal muscles were carefully removed while preserving the intervertebral ligaments, capsules, joints, and discs intact. Specimens were implanted with steel nails at L1 and S2, and embedded at L1 and S1–S2 vertically with polymethylmethacrylate (PMMA), finally wrapped with physiological saline gauze, sealed, and stored at −20°C until the flexibility test.

### Flexibility Test System

**Figure 1:**
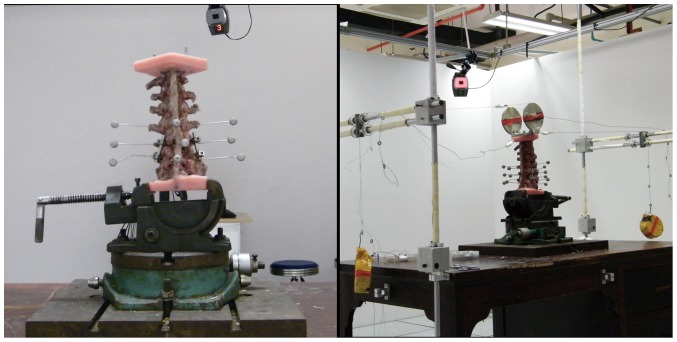
Flexibility test system. The specimen was fixed on the working table. Laser balls were used to simulate planes of representative vertebral bodies. An unstrained spinal 3-D motion was obtained by cables connected to loading discs. A suspended counterweight transferred equal but opposing forces on to the top of the specimen. The movement of the laser balls was tracked by six cameras hanging at different sites from the ceiling.

The Flexibility Test System was composed of cables, pulleys, suspended counterweights, a loading disc, and a basement. The lower side of the PMMA block was placed on the basement, the upper side of the PMMA block was fixed to the loading disc. The cables connected to the loading disc and suspended counterweight transferred equal but opposing forces on to the top of the specimen (Figure 1). Thus, the custom-made testing system could apply pure moments in flexion/extension, right/left rotation and right/left lateral bending and allowed complete, unconstrained 3-dimensional motion of the spine. To overcome the spine's viscoelastic effects, before recording motion data for each loading scenario, three preconditioning cycles were applied to the specimen, and applied moments were maintained for approximately 30 seconds. The motion of each segment was recorded using a clinical motion analysis system with four marker balls arranged rigidly along the plane of each vertebral body. ROM was calculated by Cortex software.

### Flexibility Test Protocol

Specimens were initially tested in the intact state. Subsequently, TLIF was performed; each specimen underwent a right-sided L4-5 facetectomy and discectomy. Facetectomy was performed 1 cm away from the facet surface. The superior edge of the L4 lamina, the inferior edge of the L5 lamina, and the part of the lamina connected to the vertebral pedicle were intact. Therefore, force applied to the lamina was delivered to the vertebral body under physiological conditions, and hooks instrumented on the superior edge of the L4 lamina and the inferior edge of the L5 lamina could achieve stability and resistance to failure. Each specimen was then tested for the following surgical constructs: USMAH, BSMAH, UPS, and BPS. A maximum moment of ±8 Nm was applied on all specimens as previously described [15], [17], [18]. Three-dimensional movements including flexion/extension, left/right lateral bending, and left/right axial rotation were tested on all specimens.

**Figure 2:**
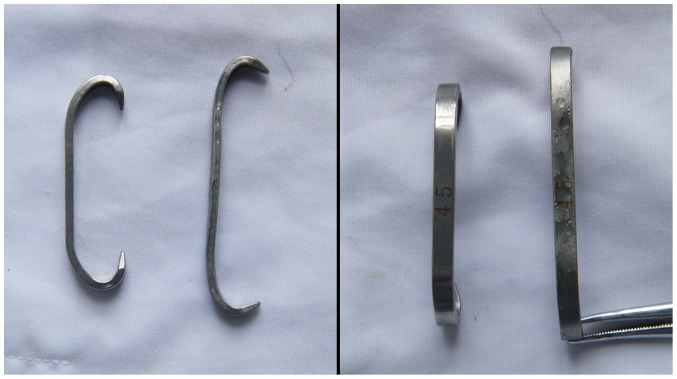
45 mm Shape memory Alloy Hook (SMAH). Left, prototype; right, unfolded in ice and water mixture.

For the USMAH, an appropriately sized TLIF spacer was inserted into the middle third of the L4-5 disc space. The length between the superior edge of the L4 lamina and inferior edge of the L5 lamina was measured to allow selection of a suitable SMAH. Different size SMAHs ranging from 39 mm to 59 mm in length were designed to meet individual needs. Every 2 mm is an interval. Therefore, if the shortest length between the superior edge of the L4 lamina and inferior edge of the L5 lamina was 45 mm, a 43 mm or 41 mm SMAH was chosen. The smaller size hook provided a persistent compressive force to stabilize the lumbar segments. After an SMAH was instrumented, stability was checked by shaking the hook. The hook was unfolded in an ice-water mixture using a stretcher, and instrumented on the right side superior edge of the L4 lamina and inferior edge of the L5 lamina using a holder (Figure 2). Gauze soaked in water at 37°C water was spread on the surface of the hook to promote re-plasticity. BSMAHs were instrumented on both side of L4 and L5 laminas, but the left facet joint was retained intact. When the SMAHs flexibility tests were complete, gauze soaked in ice-water was spread onto the surface of the hook to facilitate its removal. The pedicle screws (6.5 mm in diameter ×45 mm in length) and 5.5-mm–diameter titanium rods were instrumented according to common practice. As the SMAH did not damage the posterior structure, implantation of the pedicle screw-rod constructs was not affected by previous instrumentation with the SMAH.

**Figure 3:**
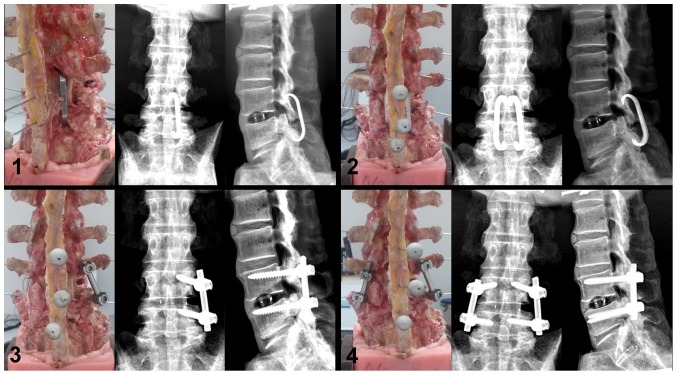
Surgical constructs. 1. Transforaminal lumbar interbody fusion (TLIF) with unilateral shape memory alloy hook (USMAH) at L4-5; 2. TLIF with bilateral shape memory alloy hook (BSMAH) at L4-5; 3. TLIF with unilateral pedicle screws and rods (UPS) at L4-5; 4. TLIF with bilateral pedicle screws and rods (BPS) at L4-5.

During the testing progress, physiological saline was sprayed on the specimen every 5 minutes to keep it moist and fresh. Testing was conducted at room temperature. X-rays were taken after each test (Figure 3). All operations were performed by an experienced spinal surgeon.

### Statistical Analysis

Statistical analyses were performed using SPSS 19.0 software. Comparison of ROM was performed using one-way analysis of variance (ANOVA) and post hoc analysis by Tukey for multiple comparison procedures. p<0.05 was considered significant.

## Results

### Range of Motion at L4-5

In flexion, all instrumented constructs significantly decreased ROM compared with the intact spine (p<0.05). BPS and BSMAH decreased ROM by 71% and 60%, respectively. UPS and USMAH decreased ROM by 41% each. There was no significant difference between the stability offered by USMAH, BSMAH, UPS and BPS.

In extension, BSMAH, UPS, and BPS significantly decreased ROM by 51%, 46%, and 70%, respectively, compared with the intact spine (p<0.05). USMAH decreased ROM by 26%; however, there was no significant difference compared with the intact spine (p>0.05). There was significant difference between the stability offered by USMAH and BPS (p<0.05).

In left lateral bending, all instrumented constructs significantly decreased ROM compared with the intact spine (p<0.05). BSMAH and BPS decreased ROM by 68% and 72%, respectively; USMAH and UPS decreased ROM by 33% and 35%, respectively. There was no significant difference between the stability offered by USMAH and UPS, or BSMAH and BPS. Bilateral fixation constructs had lower ROM compared with unilateral fixation constructs (p<0.05).

In right lateral bending, BSMAH, UPS, and BPS significantly decreased ROM compared with the intact spine (p<0.05). BSMAH and BPS decreased ROM by 52% and 71%, respectively. USMAH and UPS decreased ROM by 29% and 31%, respectively. There was no significant difference between the stability offered by USMAH and UPS, or BSMAH and BPS.

**Figure 4:**
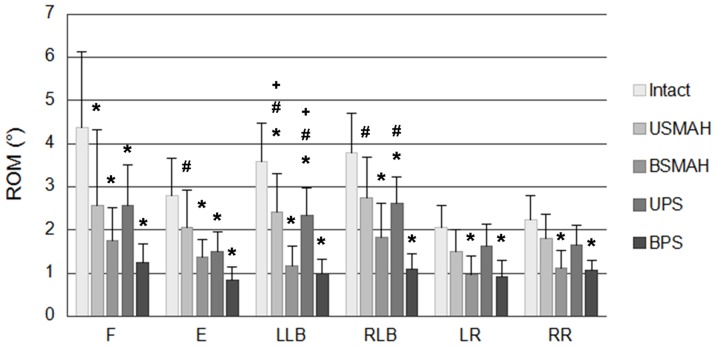
Range of motion at L4-5. Abbreviations: F, flexion; E, extension; LLB, left lateral bending; RLB, right lateral bending; LR, left rotation; RR, right rotation. USMAH, unilateral shape memory alloy hook; BSMAH, bilateral shape memory alloy hook; UPS, unilateral pedicle screws and rods; BPS, bilateral pedicle screws and rods. * significant difference vs. the intact spine (p<0.05). # significant difference vs. the BPS construct (p<0.05). + significant difference vs. the BSMAH construct (p<0.05).

In axial rotation, BSMAH and BPS significantly decreased ROM by 51% and 54%, respectively, compared with the intact spine, (p<0.05). There was no significant difference between the stability offered by USMAH and UPS compared with the intact spine (Fig. 4).

### Range of Motion at L3-4 and L5-S1

**Figure 5:**
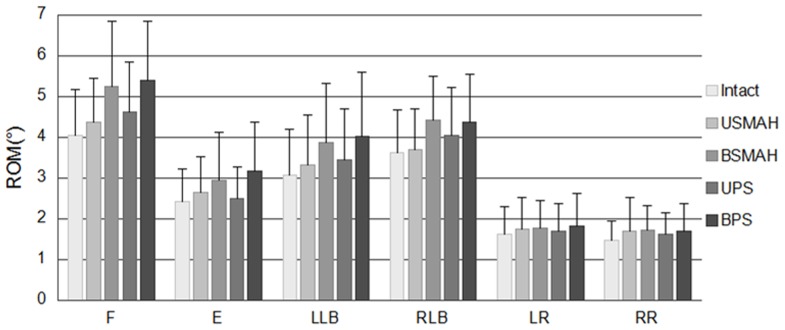
Range of motion at L3-4. Abbreviations: F, flexion; E, extension; LLB, left lateral bending; RLB, right lateral bending; LR, left rotation; RR, right rotation. USMAH, unilateral shape memory alloy hook; BSMAH, bilateral shape memory alloy hook; UPS, unilateral pedicle screws and rods; BPS, bilateral pedicle screws and rods.

**Figure 6:**
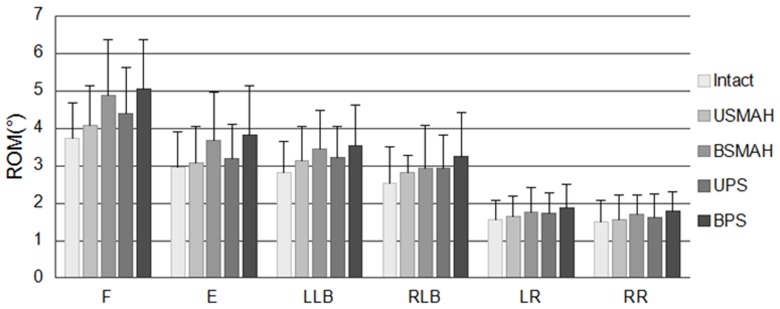
Range of motion at L5-S1. Abbreviations: F, flexion; E, extension; LLB, left lateral bending; RLB, right lateral bending; LR, left rotation; RR, right rotation. USMAH, unilateral shape memory alloy hook; BSMAH, bilateral shape memory alloy hook; UPS, unilateral pedicle screws and rods; BPS, bilateral pedicle screws and rods.

At L3-4 and L5-S1, there was no significant difference in ROM between the constructs compared with the intact spine (p>0.05). Mean ROM of BSMAH and BPS appeared higher compared with the intact spine and USMAH and UPS, but the differences were not significant (Figs. 5, 6).

## Discussion

In this study, TLIF was chosen as the model for *in vitro* spinal reconstruction and biomechanical testing. In 1998, Harms and Jeszenszky introduced TLIF as an alternative technique to PLIF in an attempt to overcome risk for damage to the conusmedullaris and dural sac [3], [19]. TLIF offers numerous theoretical and clinical advantages over PLIF, and has become more popular over the last decade. Unilateral and bilateral TLIF procedures are safe and effective options for reconstructive spinal fusion surgery, achieving lumbar fusion and relieving pain and neural compression [14], [20], [21], [22], [23]. *In vitro* biomechanical tests have demonstrated that TLIF can achieve immediate stability following surgery with unilateral or bilateral pedicle screw and rod fixation [15], [17], [24], [25]. Through a unilateral approach, TLIF can provide an adequate surface area for solid fusion as 69% disk volume (56% of endplate surface area) excision can be achieved [25]. *In vivo* analysis has revealed that 30% of the vertebral endplate surface area is the minimum required to achieve rigid interbody fusion [26]. To achieve consistent experiment conditions in this study, spacer was placed in the middle third of the L4-5 disc space, rather than the anterior third. Practically, it is easier to place a spacer in the middle third of the disc space. Most importantly the position of the spacer did not influence the maintenance of segmental lordosis or the stability of the TLIF constructs. In a finite element analysis, Chen et al demonstrated that similar ROM was achieved in TLIFa (spacer was placed in anterior third) and TLIFm(middle third) [27]. Faundez et al demonstrated that TLIFa and TLIFp(posterior third) constructs had a statistically equivalent 3-dimensional stability and maintained similar segmental lordosis. Even though diverse spacer positions have been recommended by several authors, no clinical study has shown any correlation between fusion rates and different TLIF techniques and implant positions [28].

Ni-Ti SMA instruments have a combination of good mechanical strength, specific properties such as the shape memory effect and superelasticity, and good biocompatibility [13], [29]. Therefore, Ni-Ti SMA instruments have been widely used for orthodontic tooth alignment, vascular applications, and osteosynthesis staples [20], [30]. The safty of Ni-Ti SMAs can be enhanced by alloy surface treatment [31]. Animal experiments showed that the histological response of the soft tissues around the Ni-Ti implant was clearly non-toxic and non-irritating after 26 weeks of implantation [32]. In vitro experiments demonstrated that Ni-Ti SMA instruments were completely cytocompatible and genocompatible [33]. Ni-Ti SMAs have a Young's modulus of 50 GPa. This is close to the Young's modulus of cortical bone (14 GPa), but much smaller than the Young's modulus of titanium (110 GPa). According to BO principles, an implant with a low Young's modulus can alleviate stress shielding and concentration, which can cause implant rupture and failure [34].

The Ni-Ti alloy can be bent when cooled and then retake its original shape when heated. This property, known as shape-memory, has resulted in the application of shape-memory alloy wire implantations for gradual correction of scoliosis [34], [35]. However, to the best of our knowledge, there are no publications describing shape-memory alloy fixation for lumbar fusion. Therefore, we designed the SMAH as a posterior lumber fixation system. In our cadaveric model, the SMAH implantation procedure was easy, minimally invasive, and associated with a short operation time. The hook was placed on the superior edge of the L4 lamina and inferior edge of the L5 lamina. The posterior lumbar osteostructures remained unimpaired, which may be associated with reduced blood loss compared with pedicle screw-rod constructs in clinic, and should provide perfect conditions for rebuilding and stabilization if a primary operation fails. Compared with the traditional lamina hook, the SMAH has a lower risk of hook out. Because traditional laminar hook fixation systems are composed of hooks and rods, the connection between the hook and rod may loosen, which can result in hook out. In contrast, the SMAH is an independent device. When instrumented, the SMAH applies a continuous retractive force to the lamina, which results in a reliable construct. The SMAH is also cost effective. The SMAH is not yet on the market, so the price is unknown. But the price of a similar SMA product, the Ni-Ti-patella concentrator, is 1/10 of the price of one pedicle screw and rod.

We predicted that the SMAH would be an ideal implant if it could provide enough stability after TLIF. In accordance with previous reports, our study demonstrated that the ROM of an operated segment was decreased after TLIF using unilateral or bilateral pedicle screw and rod fixation [15], [17], [24], [36], [37], but axial rotation was not well controlled by unilateral fixation. When considering the SMAH, in flexion, the USMAH and BSMAH compared favorably with the UPS and BPS. In extension, the USMAH provided less stability compared with the BSMAH and BPS. In left lateral bending, all instrumented constructs significantly restricted the lateral instability caused by right facetectomy and discectomy. However, in right lateral bending, only the BSMAH, UPS, and BPS provided stability, indicating that the USMAH could only restrict contralateral bending and supporting the recommendation for bilateral fixation. In axial rotation, only the BSMAH and BPS significantly decreased ROM. This was likely because the SMAH is positioned in opposition to the direction of motion in flexion/extension and lateral bending. In axial rotation, the hook is parallel to the axis of rotation, and the resultant limit on motion is not as great.

In right lateral bending and axial rotation, USMAH did not significantly decrease ROM compared to the intact spine; this may have been due to a small sample size. The degree of stability required to attain lumbar fusion is unknown, but it is generally believed that the ROM of an operated segment should not be larger than the intact spine. Although UPS cause a non-significant decrease in ROM in axial rotation, clinical studies have shown that TLIF with unilateral pedicle screw and rod fixation can achieve the same fusion rate and satisfaction as bilateral instrumentation [23], [38], [39]. These data suggest that implantation of the USMAH construct may result in an acceptable fusion rate and satisfactory outcomes. However, further animal and clinical studies are required to confirm this effect.

Adjacent segment degeneration (ASD) has been recognized as a potential long-term complication of rigidly instrumented fusion [40], [41], [42]. After solid fusion, there may be a change in the ROM of the spine. The ROM of adjacent segments will increase, compensating for the decreased ROM caused by a solid fixed segment [40]. To reduce the incidence of ASD, several implants of semi-rigid or dynamic stabilization of lumbar intervertebral segments have been developed. Examples of such devices are Isobar TTL, a metal rod with disc springs, the CD Horizon Legacy PEEK rod, and Dynesys Dynamic Stabilization System consisting of a polymeric dampener and posterior tensioning cord. However, most studies show the stiffness of these constructs to be too high to have much of an effect [41], [43]. We speculate that the SMAH maybe a semi-rigid fixation device for two reasons. First, the Young's modulus of Ni-Ti SMA is smaller than titanium but larger than cortical bone. A lower modulus may alleviate shielding and stress concentration. Second, the results of our study showed that SMAH constructs have a relatively higher ROM than pedicle screw and rod constructs. Therefore, SMAH fixation is probably not as rigid as fixation with pedicle screws and rods. In our study, we measured the ROM of the L3-4 and L5-S1 segments. The results showed that all implants increased the ROM of the adjacent segment, and that bilateral fixation increased the ROM to a greater extent than unilateral fixation. This indicates that a more rigid fixation resulted in an increased ROM in the adjacent segment. Notably, there was no significant difference in the ROM of the adjacent segment when compared to the intact spine. However, these results should be interpreted with caution, and the applicability of the SMAH as a semi-rigid fixation device that may be beneficial for the prevention of ASD requires further investigation. In particular, Sengupta et al. [18] demonstrated that a 10% increase in the ROM of the adjacent segment in a human cadaveric lumbosacral spine injury model implanted with a posterior dynamic stabilization device resulted in a 220% increase in intradiscal pressure compared to the intact spine. The relevance of such large pressure increases in the intradiscal and facet joint to SMAH fixation warrant consideration.

There are several limitations in our study: First, the sample size was necessarily small as we conducted our investigations using a cadaveric model. Second, our results were only indicative of immediate stabilization. Lack of experimental equipment limited our ability to apply axial pressure to simulate the physical load, and perform tests for fatigability and pull out strength. The biomechanical properties of implants after TLIF in vivo are complex. The ability of this newly developed SMAH to provide stabilization and achieve fusion *in vivo* requires further experiments and supportive clinical data. Third, we could not make a clear conclusion about the effect on the adjacent segment. These studies should be extended to include measurement of intravertebral facet-joint pressure and the use of a finite element analysis model to simulate the ROM, the intravertebral and facet-joint, and the strain distribution of the whole unit, to enable more definitive conclusions to be made. Fourth, because stability and resistance to failure of SMAH implants are dependent upon the integrity of the lamina, we performed facetectomy within 1 cm from the facet surface to ensure the lamina remained intact and was strong enough to instrument with an SMAH. However, in clinical practice, for the purpose of decompression, wide resection of lamina may be needed. Under these conditions, application of an SMAH may not be the optimal choice. Fifth, some patients have severe lumbar spinal degeneration with thickened lamina and spinal canal stenosis, which will make SMAH implantation difficult. Even if implantation is successful, epidural compression is possible. We recommend that surgeons use pre-operative CT scans to guide their decision-making, and that SMAH should be used for patients with mild osteo-degenerated lumbar diseases that require TLIF (e.g., as in discogenic back pain). We propose that the SMAH represents another option for spinal surgeons treating these diseases. Sixth, we did not test the stability of the SMAH construct when the placement of the spacer in the disc space was varied. Therefore, we plan further studies using cadaveric experiments and Finite Element Analysis.

## Conclusions

In conclusion, bilateral SMAH fixation can achieve immediate stability at L4-5 after TLIF. The newly developed SMAH is an ideal implant for lumbar fusion, with the possibility to reduce operative time and healthcare costs and improve patient outcomes. Further studies are warranted to identify the stress distribution of the whole unit and whether this fixation method could achieve an acceptable fusion rate in other segments and in vivo.
